# Global Wheat Head Detection 2021: An Improved Dataset for Benchmarking Wheat Head Detection Methods

**DOI:** 10.34133/2021/9846158

**Published:** 2021-09-22

**Authors:** Etienne David, Mario Serouart, Daniel Smith, Simon Madec, Kaaviya Velumani, Shouyang Liu, Xu Wang, Francisco Pinto, Shahameh Shafiee, Izzat S. A. Tahir, Hisashi Tsujimoto, Shuhei Nasuda, Bangyou Zheng, Norbert Kirchgessner, Helge Aasen, Andreas Hund, Pouria Sadhegi-Tehran, Koichi Nagasawa, Goro Ishikawa, Sébastien Dandrifosse, Alexis Carlier, Benjamin Dumont, Benoit Mercatoris, Byron Evers, Ken Kuroki, Haozhou Wang, Masanori Ishii, Minhajul A. Badhon, Curtis Pozniak, David Shaner LeBauer, Morten Lillemo, Jesse Poland, Scott Chapman, Benoit de Solan, Frédéric Baret, Ian Stavness, Wei Guo

**Affiliations:** ^1^Arvalis, Institut du Végétal, 3 Rue Joseph et Marie Hackin, 75116 Paris, France; ^2^UMR1114 EMMAH, INRAE, Centre PACA, Bâtiment Climat, Domaine Saint-Paul, 228 Route de l'Aérodrome, CS 40509, 84914 Avignon Cedex, France; ^3^School of Food and Agricultural Sciences, The University of Queensland, Gatton, 4343 QLD, Australia; ^4^Hiphen SAS, 120 Rue Jean Dausset, Agroparc, Bâtiment Technicité, 84140 Avignon, France; ^5^Plant Phenomics Research Center, Nanjing Agricultural University, Nanjing, China; ^6^Wheat Genetics Resource Center, Dep. of Plant Pathology, Kansas State Univ., 4024 Throckmorton Plant Sciences Center, Manhattan, Kansas, USA; ^7^Global Wheat Program, International Maize and Wheat Improvement Centre (CIMMYT), Mexico, D.F., Mexico; ^8^Faculty of Biosciences, Norwegian University of Life Sciences, P.O. Box 5003, NO-1432 Ås, Norway; ^9^Agricultural Research Corporation, Wheat Research Program, P.O. Box 126, Wad Medani, Sudan; ^10^Arid Land Research Center, Tottori University, Tottori 680-0001, Japan; ^11^Laboratories of Plant Genetics and Plant Breeding, Graduate School of Agriculture, Kyoto University, Japan; ^12^CSIRO Agriculture and Food, Queensland Biosciences Precinct, 306 Carmody Road, St Lucia, 4067 QLD, Australia; ^13^Institute of Agricultural Sciences, ETH Zurich, Universitätstrasse 2, 8092 Zurich, Switzerland; ^14^Plant Sciences Department, Rothamsted Research, Harpenden, UK; ^15^Institute of Crop Science, National Agriculture and Food Research Organization, Japan; ^16^Hokkaido Agricultural Research Center, National Agriculture and Food Research Organization, Japan; ^17^Biosystems Dynamics and Exchanges, TERRA Teaching and Research Center, Gembloux Agro-Bio Tech, University of Liège, 5030 Gembloux, Belgium; ^18^Plant Sciences, TERRA Teaching and Research Center, Gembloux Agro-Bio Tech, University of Liège, 5030 Gembloux, Belgium; ^19^Graduate School of Agricultural and Life Sciences, The University of Tokyo, 1-1-1 Midori-cho, Nishitokyo City, Tokyo, Japan; ^20^Department of Computer Science, University of Saskatchewan, Canada; ^21^Department of Plant Sciences, University of Saskatchewan, Canada; ^22^College of Agriculture and Life Sciences, University of Arizona, Tucson, Arizona, USA

## Abstract

The Global Wheat Head Detection (GWHD) dataset was created in 2020 and has assembled 193,634 labelled wheat heads from 4700 RGB images acquired from various acquisition platforms and 7 countries/institutions. With an associated competition hosted in Kaggle, GWHD_2020 has successfully attracted attention from both the computer vision and agricultural science communities. From this first experience, a few avenues for improvements have been identified regarding data size, head diversity, and label reliability. To address these issues, the 2020 dataset has been reexamined, relabeled, and complemented by adding 1722 images from 5 additional countries, allowing for 81,553 additional wheat heads. We now release in 2021 a new version of the Global Wheat Head Detection dataset, which is bigger, more diverse, and less noisy than the GWHD_2020 version.

## 1. Introduction

Quality training data is essential for the deployment of deep learning (DL) techniques to get a general model that can scale on all the possible cases. Increasing dataset size, diversity, and quality is expected to be more efficient than increasing network complexity and depth [1]. Datasets like ImageNet [2] for classification or MS COCO [3] for instance detection are crucial for researchers to develop and rigorously benchmark new DL methods. Similarly, the importance of getting plant- or crop-specific datasets is recognized within the plant phenotyping community ([4–10], p. 2, [11–13]). These datasets allow benchmarking the algorithm performances used to estimate phenotyping traits while encouraging computer vision experts to further improvement ([10], p. 2, [14–17]). The emergence of affordable RGB cameras and platforms, including UAVs and smartphones, makes in-field image acquisition easily accessible. These high-throughput methods are progressively replacing manual measurement of important traits such as wheat head density. Wheat is a crop grown worldwide, and the number of heads per unit area is one of the main components of yield potential. Creating a robust deep learning model performing over all the situations requires a dataset of images covering a wide range of genotypes, sowing density and pattern, plant state and stage, and acquisition conditions. To answer this need for a large and diverse wheat head dataset with consistent and quality labeling, we developed in 2020 the Global Wheat Head Detection (GWHD_2020) [18] that was used to benchmark methods proposed in the computer vision community and recommend best practices to acquire images and keep track of the metadata.

The GWHD_2020 dataset results from the harmonization of several datasets coming from nine different institutions across seven countries and three continents. There are already 27 publications [19–45] (accessed July 2021) that have reported their wheat head detection model using the GWHD_2020 dataset as the standard for training/testing data. A “Global Wheat Detection” competition hosted by Kaggle was also organized, attracting 2245 teams across the world [14], leading to improvements in wheat head detection models [23, 25, 31, 41]. However, issues with the GWHD_2020 dataset were detected during the competition, including labeling noise and an unbalanced test dataset.

To provide a better benchmark dataset for the community, the GWHD_2021 dataset was organized with the following improvements: (1) the GWHD_2020 dataset was checked again to eliminate few poor-quality images, (2) images were re-labeled to avoid consistency issues, (3) a wider range of developmental stages from the GWHD_2020 sites was included, and (4) datasets from 5 new countries (the USA, Mexico, Republic of Sudan, Norway, and Belgium) were added. The resulting GWHD_2021 dataset contains 275,187 wheat heads from 16 institutions distributed across 12 countries.

## 2. Materials and Methods

The first version of GWHD_2020, used for the Kaggle competition, was divided into several subdatasets. Each subdataset represented all images from one location, acquired with one sensor while mixing several stages. However, wheat head detection models may be sensitive to the developmental stage and acquisition conditions: at the beginning of head emergence, a part of the head is barely visible because it is still not fully out from the last leaf sheath and possibly masked by the awns. Further, during ripening, wheat heads tend to bend and overlap, leading to more erratic labeling. A redefinition of the subdataset was hence necessary to help investigate the effect of the developmental stage on model performances. The new definition of a subdataset was then formulated as “a consistent set of images acquired over the same experimental unit, during the same acquisition session with the same vector and sensor.” A subdataset defines therefore a domain. This new definition forced to split the original GWHD_2020 subdatasets into several smaller ones. The UQ_1 was split into 6 much smaller subdatasets, Arvalis_1 was split into 3 subdatasets, Arvalis_3 into 2 subdatasets, and utokyo_1 into 2 subdatasets. However, in the case of utokyo_2 which was a collection of images taken by farmers at different stages and in different fields, the original subdataset was kept. Overall, the 11 original subdatasets in GWHD_2020 were distributed into 19 subdatasets for GWHD_2021.

Almost 2000 new images were added to GWHD_2020, constituting a major improvement. A part of the new images comes from the institutions already contributing to GWHD_2020 and was collected during a different year and/or at a different location. This was the case for Arvalis (Arvalis_7 to Arvalis_12), University of Queensland (UQ_7 to UQ_11), Nanjing Agricultural University (NAU_2 and NAU_3), and University of Tokyo (Utokyo_1). In addition, 14 new subdatasets were included, coming from 5 new countries: Norway (NMBU), Belgium (Université of Liège [46]), United States of America (Kansas State University [47], TERRA-REF [7]), Mexico (CIMMYT), and Republic of Sudan (Agricultural Research Council). All these images were acquired at a ground sampling distance between 0.2 and 0.4 mm, i.e., similar to that of the images in GWHD_2020. Because none of them was already labeled, a sample was selected by taking no more than one image per microplot, which was randomly cropped to 1024 × 1024 px patches that will be called images in the following for the sake of simplicity.

**Figure 1:**
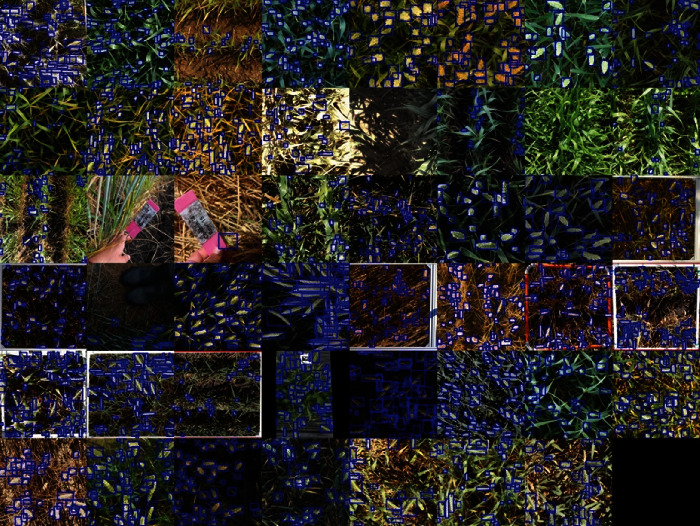
Sample images of the Global Wheat Head Detection 2021. The blue boxes correspond to the interactively labeled heads.

**Figure 2:**
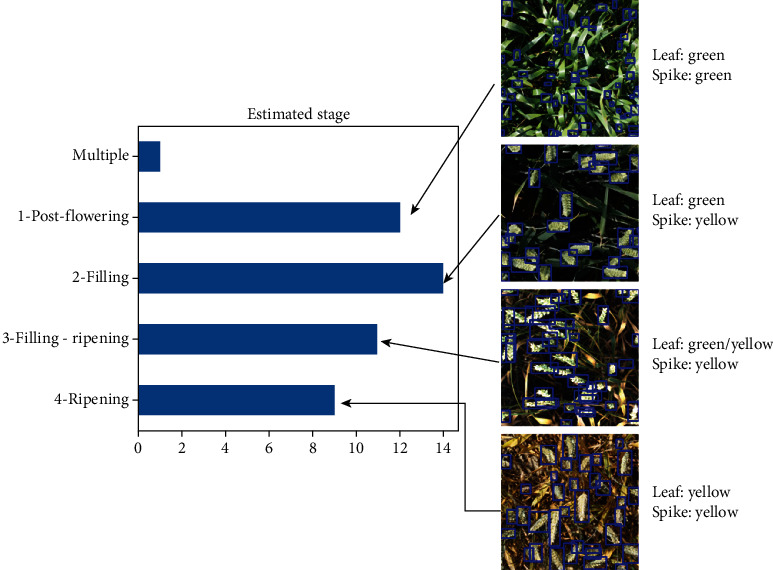
Distribution of the development stage. The *x*-axis presents the number of subdataset per development stage.

With the addition of 1722 images and 86,000 wheat heads, the GWHD_2021 dataset contains 6500 images and 275,000 wheat heads. The increase in the number of subdatasets from 18 to 47 leads to a larger diversity between them which can be observed on Figure 1. The subdatasets are described in Table 1. However, the new definition of a subdataset led also to more unbalanced subdatasets: the smallest (Arvalis_8) contains only 20 images, while the biggest (ETHZ_1) contains 747 images. This provides the opportunity to possibly take advantage of the data distribution to improve model training. Each subdataset has been visually assigned to several development stage classes depending on the respective color of leaves and heads (Figure 2): postflowering, filling, filling-ripening, and ripening. Examples of the different stages are presented in Figure 2. While being approximative, this metadata is expected to improve model training.

## 3. Dataset Diversity Analysis

In comparison to GWHD_2020, the GWHD_2021 dataset puts emphasis on metadata documentation of the different subdatasets, as described in the discussion section of David et al. [18]. Alongside the acquisition platform, each subdataset has been reviewed and a development stage was assigned to each, except for Utokyo_3 (formerly utokyo_2) as it is a collection of images from various farmer fields and development stages. Globally, the GWHD_2021 dataset covers well all development stages ranging from postanthesis to ripening (Figure 2).

**Figure 3:**
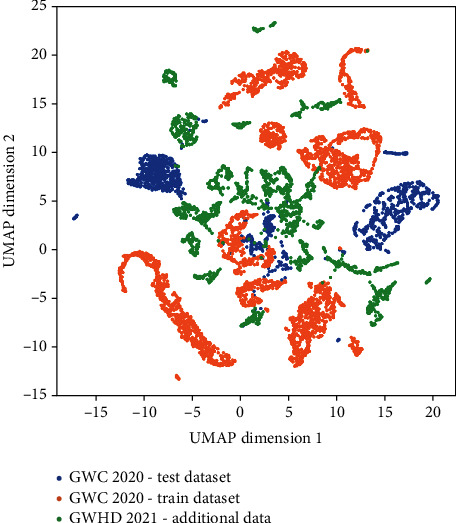
Distribution of the images in the two first dimensions defined by the UMAP algorithm for the GWHD 2021 dataset. The additional subdatasets as well as the training and test datasets from GWHD_2020 are represented by colors.

The diversity between images within the GWHD_2021 dataset was documented using the method proposed by Tolias et al. [48]. The deep learning image features were first extracted from the VGG-16 deep network pretrained on the ImageNet dataset that is considered representing well the general features of RGB images. We then selected the last layer which has a size of 14 × 14 × 512 and summed it into a unique vector of 512 channels, which is then normalized. Then, the UMAP dimentionality reduction algorithm [49] was used to project representations into a 2D space. The UMAP algorithm is used to keep the existing clusters during the projection to a low-dimension space. This 2D space is expected to capture the main features of the images. Results (Figure 3) demonstrate that the test dataset used for GWHD_2020 was biased in comparison to the training dataset. The subdatasets added in 2021 populate more evenly the 2D space which is expected to improve the robustness of the models.

## 4. Presentation of Global Wheat Challenge 2021 (GWC 2021)

The results from the Kaggle challenge based on GWHD_2020 have been analyzed by the authors [14]. The findings emphasize that the design of a competition is critical to enable solutions that improve the robustness of the wheat head detection models. The Kaggle competition was based on a metric that was averaged across all test images, without distinction for the subdatasets, and it was biased toward a strict match of the labelling. This artificially enhances the influence on the global score of the largest datasets such as utokyo_1 (now split into Utokyo_1 and Utokyo_2). Further, the metrics used to score the agreement with the labeled heads and largely used for big datasets, such as MS COCO, appear to be less efficient when some heads are labeled in a more uncertain way as it was the case in several situations depending on the development stage, illumination conditions, and head density. As a result, the weighted domain accuracy is proposed as a new metric [14]. The accuracy computed over image *i* belonging to domain *d*, AI_*d*_(*i*), is classically defined as

(1) $$\begin{array}{c} {\text{AI}}_{d}\left(i\right)=\frac{\text{TP}}{\text{TP}+\text{FN}+\text{FP}}, \end{array}$$

where TP, FN, and FP are, respectively, the number of true positive, false negative, and false positive found in image *i*. The weighted domain accuracy (WDA) is the weighted average of all domain accuracies:

(2) $$\begin{array}{c} \text{WDA}=\frac{1}{D}\overset{D}{\underset{d=1}{\sum }}\frac{1}{n_{d}}*\overset{n_{d}}{\underset{i=1}{\sum }}{\text{AI}}_{di}, \end{array}$$

where *D* is the number of domains (subdatasets) and *n*_*d*_ is the number of images in domain *d*. The training, validation, and test datasets used are presented in Section 5.

The results of the Global Wheat Challenge 2021 are summarized in Table 2. The reference method is a faster-RCN with the same parameters than in the research paper GWHD_2020 [18] and trained on the GWHD_2021 (Global Wheat Challenge 2021 split) training dataset. The full leaderboard can be found at https://www.aicrowd.com/challenges/global-wheat-challenge-2021/leaderboards.

## 5. How to Use/FAQ

- How to download? The dataset can be download on Zenodo: https://zenodo.org/record/5092309
- What is the license of the dataset? The dataset is under the MIT license, allowing for reuse without restriction
- How to cite the dataset? The present paper can be cited when using the GWHD_2021 dataset. However, cite preferentially [18] for wheat head detection challenges or when discussing the difficulty to constitute a large datasets
- How to benchmark? Depending on the objectives of the study, we recommend two sets of training, validation, and test (Table 3):

- The Global Wheat Challenge 2021 split when the dataset is used for phenotyping purpose, to allow direct comparison with the winning solutions
- The “GlobalWheat-WILDS” split is the one used for the WILDS paper [50]. We recommand to use the GlobalWheat-WILDS split when working on out-of-domain distribution shift problems

It is further recommended to keep the weighted domain accuracy for comparison with previous works.

## 6. Conclusion

The second edition of the Global Wheat Head Detection, GWHD_2021, alongside the organization of a second Global Wheat Challenge is an important step for illustrating the usefulness of open and shared data across organizations to further improve high-throughput phenotyping methods. In comparison to the GWHD_2020 dataset, it represents five new countries, 22 new subdatasets, 1200 new images, and 120,000 new-labeled wheat heads. Its revised organization and additional diversity are more representative of the type of images researchers and agronomists can acquire across the world. The revised metrics used to evaluate the models during the Global Wheat Challenge 2021 can help researchers to benchmark one-class localization models on a large range of acquisition conditions. GWHD_2021 is expected to accelerate the building of robust solutions. However, progress on the representation of developing countries is still expected and we are open to new contributions from South America, Africa, and South Asia. We started to include nadir view photos from smartphones, to get a more comprehensive dataset and train reliable models for such affordable devices. Additional works are required to adapt such an approach to other vectors such as a camera mounted on unmanned aerial vehicle, or other high-resolution cameras working in other spectral domains. Further, it is planned to release wheat head masks alongside the bounding box given the very large number of boxes that already exists and provides more associated metadata.

## Figures and Tables

**Table 1:** The subdatasets for GWHD_2020 and GWHD_2021. The column “2020 name” indicates the name given to the subdatasets for GWHD_2020, which were split into several new subdatasets.

GWHD_2021 subdataset name	GWHD_2020 subdataset name	Owner	Country	Location	Acquisition date	Platform	Development stage	Number of images	Number of wheat head
*Ethz_1*	*ethz_1*	*ETHZ*	*Switzerland*	*Usask*	*06/06/2018*	*Spidercam*	*Filling*	*747*	*49603*
*Rres_1*	*rres_1*	*Rothamsted*	*UK*	*Rothamsted*	*13/07/2015*	*Gantry*	*Filling-ripening*	*432*	*19210*
*ULiège-GxABT_1*		*Uliège/Gembloux*	*Belgium*	*Gembloux*	*28/07/2020*	*Cart*	*Ripening*	*30*	*1847*
*NMBU_1*		*NMBU*	*Norway*	*NMBU*	*24/07/2020*	*Cart*	*Filling*	*82*	*7345*
*NMBU_2*		*NMBU*	*Norway*	*NMBU*	*07/08/2020*	*Cart*	*Ripening*	*98*	*5211*
*Arvalis_1*	*arvalis_1*	*Arvalis*	*France*	*Gréoux*	*02/06/2018*	*Handheld*	*Postflowering*	*66*	*2935*
*Arvalis_2*	*arvalis_1*	*Arvalis*	*France*	*Gréoux*	*16/06/2018*	*Handheld*	*Filling*	*401*	*21003*
*Arvalis_3*	*arvalis_1*	*Arvalis*	*France*	*Gréoux*	*07/2018*	*Handheld*	*Filling-ripening*	*588*	*21893*
*Arvalis_4*	*arvalis_2*	*Arvalis*	*France*	*Gréoux*	*27/05/2019*	*Handheld*	*Filling*	*204*	*4270*
*Arvalis_5*	*arvalis_3*	*Arvalis*	*France*	*VLB* ^∗^	*06/06/2019*	*Handheld*	*Filling*	*448*	*8180*
*Arvalis_6*	*arvalis_3*	*Arvalis*	*France*	*VSC* ^∗^	*26/06/2019*	*Handheld*	*Filling-ripening*	*160*	*8698*
*Arvalis_7*		*Arvalis*	*France*	*VLB* ^∗^	*06/2019*	*Handheld*	*Filling-ripening*	*24*	*1247*
*Arvalis_8*		*Arvalis*	*France*	*VLB* ^∗^	*06/2019*	*Handheld*	*Filling-ripening*	*20*	*1062*
*Arvalis_9*		*Arvalis*	*France*	*VLB* ^∗^	*06/2020*	*Handheld*	*Ripening*	*32*	*1894*
*Arvalis_10*		*Arvalis*	*France*	*Mons*	*10/06/2020*	*Handheld*	*Filling*	*60*	*1563*
*Arvalis_11*		*Arvalis*	*France*	*VLB* ^∗^	*18/06/2020*	*Handheld*	*Filling*	*60*	*2818*
*Arvalis_12*		*Arvalis*	*France*	*Gréoux*	*15/06/2020*	*Handheld*	*Filling*	*29*	*1277*
*Inrae_1*	*inrae_1*	*INRAe*	*France*	*Toulouse*	*28/05/2019*	*Handheld*	*Filling-ripening*	*176*	*3634*
Usask_1	**usask_1**	**USaskatchewan**	**Canada**	**Saskatchewan**	**06/06/2018**	**Tractor**	**Filling-ripening**	**200**	**5985**
KSU_1		**Kansas State University**	**US**	**KSU**	**19/05/2016**	**Tractor**	**Postflowering**	**100**	**6435**
KSU_2		**Kansas State University**	**US**	**KSU**	**12/05/2017**	**Tractor**	**Postflowering**	**100**	**5302**
KSU_3		**Kansas State University**	**US**	**KSU**	**25/05/2017**	**Tractor**	**Filling**	**95**	**5217**
KSU_4		**Kansas State University**	**US**	**KSU**	**25/05/2017**	**Tractor**	**Ripening**	**60**	**3285**
Terraref_1		**TERRA-REF project**	**US**	**Maricopa, AZ**	**02/04/2020**	**Gantry**	**Ripening**	**144**	**3360**
Terraref_2		**TERRA-REF project**	**US**	**Maricopa, AZ**	**20/03/2020**	**Gantry**	**Filling**	**106**	**1274**
CIMMYT_1		**CIMMYT**	**Mexico**	**Ciudad Obregon**	**24/03/2020**	**Cart**	**Postflowering**	**69**	**2843**
CIMMYT_2		**CIMMYT**	**Mexico**	**Ciudad Obregon**	**19/03/2020**	**Cart**	**Postflowering**	**77**	**2771**
CIMMYT_3		**CIMMYT**	**Mexico**	**Ciudad Obregon**	**23/03/2020**	**Cart**	**Postflowering**	**60**	**1561**
Utokyo_1	utokyo_1	UTokyo	Japan	NARO-Tsukuba	22/05/2018	Cart ${}^{\underset{¯}{**}}$	Ripening	538	14185
Utokyo_2	utokyo_1	UTokyo	Japan	NARO-Tsukuba	22/05/2018	Cart ${}^{\underset{¯}{**}}$	Ripening	456	13010
Utokyo_3	utokyo_2	UTokyo	Japan	NARO-Hokkaido	Multi-years ${}^{\underset{¯}{***}}$	Handheld	Multiple	120	3085
Ukyoto_1		UKyoto	Japan	Kyoto	30/04/2020	Handheld	Postflowering	60	2670
NAU_1	NAU_1	NAU	China	Baima	n.a	Handheld	Postflowering	20	1240
NAU_2		NAU	China	Baima	02/05/2020	Cart	Postflowering	100	4918
NAU_3		NAU	China	Baima	09/05/2020	Cart	Filling	100	4596
*UQ_1*	***uq_1***	***UQueensland***	***Australia***	***Gatton***	***12/08/2015***	***Tractor***	***Postflowering***	***22***	***640***
*UQ_2*	***uq_1***	***UQueensland***	***Australia***	***Gatton***	***08/09/2015***	***Tractor***	***Postflowering***	***16***	***39***
*UQ_3*	***uq_1***	***UQueensland***	***Australia***	***Gatton***	***15/09/2015***	***Tractor***	***Filling***	***14***	***297***
*UQ_4*	***uq_1***	***UQueensland***	***Australia***	***Gatton***	***01/10/2015***	***Tractor***	***Filling***	***30***	***1039***
*UQ_5*	***uq_1***	***UQueensland***	***Australia***	***Gatton***	***09/10/2015***	***Tractor***	***Filling-ripening***	***30***	***3680***
*UQ_6*	***uq_1***	***UQueensland***	***Australia***	***Gatton***	***14/10/2015***	***Tractor***	***Filling-ripening***	***30***	***1147***
*UQ_7*		***UQueensland***	***Australia***	***Gatton***	***06/10/2020***	***Handheld***	***Ripening***	***17***	***1335***
*UQ_8*		***UQueensland***	***Australia***	***McAllister***	***09/10/2020***	***Handheld***	***Ripening***	***41***	***4835***
*UQ_9*		***UQueensland***	***Australia***	***Brookstead***	***16/10/2020***	***Handheld***	***Filling-ripening***	***33***	***2886***
*UQ_10*		***UQueensland***	***Australia***	***Gatton***	***22/09/2020***	***Handheld***	***Filling-ripening***	***53***	***8629***
*UQ_11*		***UQueensland***	***Australia***	***Gatton***	***31/08/2020***	***Handheld***	***Postflowering***	***42***	***4345***
ARC_1		**ARC**	**Sudan**	**Wad Medani**	**03/2021**	**Handheld**	**Filling**	**30**	**888**
							Total	6515	275187

^∗^VLB: Villiers le Bâcle; VSC: Villers-Saint-Christophe. ^∗∗^Utokyo_1 and Utokyo_2 were taken at the same location with different sensors. ^∗∗∗^Utokyo_3 is a special subdataset made from images coming from a large variety of farmers in Hokaido between 2016 and 2019. Italic: Europe: bold: North America; underline: Asia; bold italic: Oceania; bold underline: Africa.

**Table 2:** Presentation of the Global Wheat Challenge 2021 results.

Solution name	WDA
randomTeamName (1^st^ place)	0.700
David_jeon (2^nd^ place)	0.695
SMART (2^nd^ place)	0.695
Reference (faster-RCNN)	0.492

**Table 3:** Presentation of the different splits which can be used with the GWHD_2021.

Splits	Training	Validation	Test
Global Wheat Challenge 2021	Ethz_1, Rres_1, Inrae_1, Arvalis (all), NMBU (all), ULiège-GxABT (all)	UQ_1 to UQ_6, Utokyo (all), NAU_1, Usask_1	UQ_7 to UQ_12, Ukyoto_1, NAU_2 and NAU_3, ARC_1, CIMMYT (all), KSU (all), Terraref (all)
GlobalWheat-WILDS	Ethz_1, Rres_1, Inrae_1, Arvalis (all), NMBU (all), ULiège-GxABT (all)	UQ (all), Utokyo (all), Ukyoto_1, NAU (all)	CIMMYT (all), KSU (all); Terraref (all), Usask_1, ARC_1

## Data Availability

The dataset is available on Zenodo (https://zenodo.org/record/5092309).
